# We are being blacked out, but light is within us!

**DOI:** 10.1080/19336934.2022.2162603

**Published:** 2022-12-28

**Authors:** Iryna Kozeretska

**Affiliations:** National Antarctic Scientific Centre of Ukraine, Taras Shevchenko Blvd, Kyiv, Ukraine

Each person has his or her own creative path, imbued with various ideas and their implementation. But, at one moment all plans can change abruptly, and in our case that happened due to the war that began 8 years ago and reached full scale starting on February 24^th^, 2022. Yes, we had been officially warned by foreign politicians that a big war might break out in our country, but accepting this fact was extremely hard not only for scientists, but for the broader public as well.

Several groups of *Drosophila* researchers work in Ukraine at various scientific institutions and universities, such as V. N. Karazin Kharkiv National University; Taras Shevchenko National University of Kyiv; Ivan Franko National University of Lviv; Odesa I. I. Mechnikov National University ; Vasyl Stefanyk Precarpathian National University in Ivano-Frankivsk. The working conditions of different researchers who use fruit flies are similar – most of them teach, so changing to the online teaching mode has become the general ‘norm’ for all. Each of us has received an extra pile of problems organizing our work, as from now on we have to pay attention to the endless wailing of air raid sirens, bombing and shelling, blackouts, disruption of the internet and mobile connections, and funding cuts. We have to be ready at any moment to change our plans, depending on a situation that is beyond our control.

Moreover, almost all of us have a loved one on the front line: father, husband, son, daughter, brother, or sister. Our thoughts and hearts are always with them, and it is very challenging to focus on work when we receive disturbing news, or even worse – no news for several days. But we seek inspiration and strength for life in work, in our favourite research model object. We continue our work and our research!

24 February 2022, however, did introduce significant differences for the universities depending on their location. On the very first day of the war, Kharkiv became a front-line city in the war zone.

**Kharkiv**

At that moment at Kharkiv University, active work was undertaken to maintain the ‘Collection of *Drosophila* stocks’ – part of the National Heritage of Ukraine. Moreover, personal collections of Kharkiv scientists and ongoing student projects were threatened (Bachelor’s thesis defences had been scheduled for June 2022, Master’s thesis defences had been scheduled for December 2022). Very soon they realized that it was dangerous to go to the laboratory, and it was almost impossible to get there from suburban areas. There were long queues of cars leaving the city, buses were stuck in traffic jams, and it was almost impossible to get fuel. But the flies!!! Denis Grygoryev (a Senior Laboratory Assistant at the Department of Genetics and Cytology at V. N. Karazin Kharkiv National University) went to the laboratory on foot and prepared media and dishes for passaging flies. Bombing on March 1^st^ caused structural damage to the University building, and leaving broken windows in the auditoria facing the Kharkiv Regional State Administration and the Freedom Square. Luckily, the *Drosophila* laboratory faced the backyard, there was electricity and water and there was a large thermostat for keeping the National Heritage collection. Denis kept on going to the university on foot and continued to work. New day – new challenge: the heating and electricity shut down occasionally, so the thermostat could not always keep up with the workload and Denis eventually took the *Drosophila* collection home. Student projects were set to ’pending’ or just terminated. In May, when the situation improved, the flies were returned to the university. Nowadays, Nadiia Filiponenko, also a Senior Laboratory Assistant at the Department of Genetics and Cytology of V. N. Karazin Kharkiv National University, is taking care of them.

## Kyiv

At the beginning of the war, active hostilities also took place in the Kyiv region. My own research group worked in Kyiv within the framework of the European *Drosophila* Population Genomics Consortium. Svitlana Serga, Oleksandra Protsenko and Nadiia Pirko (all of them Assistant Professors at Taras Shevchenko National University) were evacuated with their children during the heavy bombardments, respectively to France, Germany, and Switzerland. I, being Deputy Director for Scientific Work at the National Antarctic Scientific Center of Ukraine, did not leave Ukraine. However, I was evacuated for 1.5 months to the western part of the country, which was less affected by the war. Pavlo Kovalenko (PhD student) was on an expedition to the Antarctic at the Akademik Vernadsky station during the hostilities in the Kyiv region. Svitlana Serga continued research in collaboration with Nicolas Rode and Arnaud Estoup at the Center for Biology and Management of Populations (CBGP, INRAE, Montpellier, France). Her project on the endosymbiotic bacteria *Wolbachia* in *Drosophila suzukii* was supported by two funding grants from the PAUSE program (PAUSE – Solidarity with Ukraine and PAUSE ANR Gandhi-Ukraine). Thanks to this support, Svitlana has been able to continue her research in an environment that is safe for her and her children. Oleksandra Protsenko does not have the opportunity to work in Germany and therefore is temporarily not engaged in scientific research. Nadiia Pirko, together with a collection of isofemale fly lines from natural populations of Ukraine, was evacuated to Switzerland and accepted by EPFL (Lausanne) to the Laboratory of *Drosophila* Immunity led by Bruno Lemaitre. Later, the isofemale lines from natural populations of Ukraine were transferred to CBGP (INRAE, Montpellier, France) where Svitlana Serga is working, because Nadiia Pirko had to return to Ukraine to take care of her mother and sister who had been evacuated from Mariupol. We are deeply grateful to Bruno Lemaitre, Nicolas Rode, and Arnaud Estoup for kindly hosting Ukrainian scientists, providing them with a workplace, and helping to save the collection of Ukrainian flies.

## Odesa

Denis Radionov (Associate Professor), Svitlana Belokon (Associate Professor) and Tetyana Alieksieieva (Associate Professor) from Odesa I. I. Mechnikov National University, who are also part of the European *Drosophila* Population Genomics Consortium, were not able to perform their seasonal collection of local *Drosophila melanogaster* samples because Air Defence units were deployed at their traditional collection site. In the middle of summer, the forces were relocated, and they were able to renew their collection and analysis of the natural fly population of the Odesa region. This work was also conducted in the autumn of 2022. Thanks to help from members of the Department of Genetics and Molecular Biology, they managed to preserve their collection of flies. Nataliia Streltsova kept and cared for the *Drosophila* collections during the most dangerous times.

Denis Radionov is also a biology teacher at the Odesa private high-school ‘Mriya’. Under his supervision, the students there have participated in the international project ‘Melanogaster: Catch the Fly!’ (#MelanogasterCTF) for three years. The project is one of the 3 selected to be part of the prestigious 2022 annual event of the European Research Council (ERC) ‘Citizen Science and Frontier Science’, which took place on December 7^th^, 2022, at the ERC headquarters in Brussels. The ‘Mriya’ group is one of the two schools selected to present their citizen-science project at the ERC event. Unfortunately, because of the war, the students were not allowed to go to the countryside to collect fly samples themselves (though they were willing to do so), so Denis Radionov did it himself and the students analysed the collected samples later.

## Ivano-Frankivsk

At Vasyl Stefanyk Precarpathian National University all experiments with living fruit flies were interrupted and researchers concentrated only on frozen samples while still taking care of the available strains. After February 24^th^, most of the team’s time was dedicated to publishing data collected prior to the full-scale invasion by Russia. During that time, the scientists finished the revision of a manuscript about lifespan extension in fruit flies by chilli pepper, while another one, published in the journal *Redox Report*, was written from scratch during wartime. 10 October 2022 brought new troubles, when the electricity at the department was cut off, and a gasoline generator had to be set up in order to keep freezers working. At present, there is very little time for work or even for answering emails. Those two to five hours a day that we have electricity we try to use to do multiple lab maintenance chores.

## Lviv

On February 24^th^, the administration of Ivan Franko National University of Lviv made the only possible decision at that time – to transfer all work to remote mode with an immediate ban on employees coming to the workplace. And, a few days later, saving the university museum’s *Drosophila* lines was added the already existing anxiety about life, home, and loved ones on the battlefield. *Drosophila* is a unique model object, and it does not forgive laziness, chaos, disorganization, or the depredations of war. The local researchers, nonetheless, managed to preserve their entire museum, which contains about 300 fly strains, including morphological mutants for student educational programmes and special strains for studying ageing and neurodegeneration. Thanks to efforts by Nataliya Matiytsiv (Associate Professor) and engineers Marta Tkachuk and Chrystyna Havryshkevych, the entire programme of experimental work could be resumed in the laboratory in July. Since September, they have even been conducting laboratory classes for students.

This year, flies from Ukrainian natural populations have been collected at 5 localities. The samples from the Odesa and Kyiv populations were collected during air-raid alerts and actual bombing raids. We did not have the opportunity to establish isofemale lines this year. It is also important to mention the problems with the preservation of reagents and biological samples in freezers and the impossibility of conducting molecular biological research due to constant power outages. Even though the most active and the most devastating phase of the war started on February 24^th^, the conflict has been going on since 2014, starting with the annexation of Crimea and the invasion of the Donbas region by Russian-backed forces. The events of 2014 affected the well-being of the entire country and led to reductions in financial support for research. Research output from the occupied regions declined, as it became impossible to collect samples in many areas, such as Crimea. In the opinion of Ukrainians, the current war is an attempt to erase their country from the face of the Earth, sack their scientific and cultural heritage, and destroy any hope of an independent identity.

In these challenging times, we are grateful to the global scientific community, which stands together with Ukrainian scientists, supports us morally, and creates financial support mechanisms to preserve and develop science in Ukraine. We are grateful to Ukrainian scientists from across world for supporting their home country by helping us with laboratory supplies, organizing events, and hosting our colleagues in the darkest times.

We continue to work and publish, present at conferences, and conduct internships. Using this opportunity, we would like to invite everyone to join the ‘Drosophila in Experimental Genetics and Biology’ conference, which takes place biannually and will be held in Ivano-Frankivsk in 2023 in a mixed format. You can find more information at https://kbb.pnu.edu.ua/en/drosophila-conference-2023-2/.

Of course, the war will have significant long-term consequences for our scientific activity. BUT,

- Where a group of drosophilists works, there will always be *Drosophila*!
- We are being blacked out, but light is inside us!
- Life and scientific work continue even during wartime.

**We owe the fact that we are alive and can continue *Drosophila* research to the really courageous defenders of Ukraine! Slava Ukraini!**

